# Fibroblast growth factor (FGF) signaling regulates transforming growth factor beta (TGF*β*)-dependent smooth muscle cell phenotype modulation

**DOI:** 10.1038/srep33407

**Published:** 2016-09-16

**Authors:** Pei-Yu Chen, Lingfeng Qin, Guangxin Li, George Tellides, Michael Simons

**Affiliations:** 1 Yale Cardiovascular Research Center, Department of Internal Medicine, Yale University School of Medicine, New Haven, CT, USA; 2 Department of Surgery, Yale University School of Medicine, New Haven, CT, USA; 3 Department of Vascular Surgery, The First Hospital of China Medical University, 155 Nanjing Bei Street, Shenyang, China; 4 Department of Cell Biology, Yale University School of Medicine, New Haven, CT, USA

## Abstract

Smooth muscle cells (SMCs) in normal blood vessels exist in a highly differentiate state characterized by expression of SMC-specific contractile proteins (“contractile phenotype”). Following blood vessel injury *in vivo* or when cultured *in vitro* in the presence of multiple growth factors, SMC undergo a phenotype switch characterized by the loss of contractile markers and appearance of expression of non-muscle proteins (“proliferative phenotype”). While a number of factors have been reported to modulate this process, its regulation remains uncertain. Here we show that induction of SMC FGF signaling inhibits TGF*β* signaling and converts contractile SMCs to the proliferative phenotype. Conversely, inhibition of SMC FGF signaling induces TGF*β* signaling converting proliferating SMCs to the contractile phenotype, even in the presence of various growth factors *in vitro* or vascular injury *in vivo*. The importance of this signaling cross-talk is supported by *in vivo* data that show that an SMC deletion of a pan-FGF receptor adaptor *Frs2α* (fibroblast growth factor receptor substrate 2 alpha) in mice profoundly reduces neointima formation and vascular remodelling following carotid artery ligation. These results demonstrate that FGF-TGF*β* signaling antagonism is the primary regulator of the SMC phenotype switch. Manipulation of this cross-talk may be an effective strategy for treatment of SMC-proliferation related diseases.

Vascular smooth muscle cells (SMCs) are specialized blood vessel cells that play an important role in regulation of blood vessel tone, pressure, and flow. In a mature normal blood vessel, vascular SMCs exhibit a contractile (differentiated) phenotype characterized by expression of contractile markers, such as smooth muscle *α*-actin (SM *α*-actin), smooth muscle 22 alpha (SM22*α*), smooth muscle calponin, and smooth muscle myosin heavy chain (SM-MHC). In response to a vessel injury, mechanical stresses or when cultured in the presence of serum, SMCs undergo a profound transformation to a less differentiated state. This state, referred to as a synthetic phenotype, is characterized by decreased expression of contractile proteins and increased expression of non-smooth muscle proteins. The end result of such a phenotype change is increased SMC proliferative and migratory behavior and enhanced deposition of the extracellular matrix^1^,^2^. While the phenotypic modulation of vascular SMCs is an important vascular injury repair mechanism, it also plays a major role in the pathogenesis of a number of diseases, including atherosclerosis, restenosis and transplant vasculopathy among others. Indeed, a number of therapies have sought to target this process with the view towards reversing pathologies associated with these diseases. Despite much work that has been focused on identification of the molecular mechanisms involved in SMC phenotype modulation, many aspects of this phenomenon remain poorly understood.

A number of factors involved in regulation of SMC phenotype plasticity have been identified. Among these, platelet-derived growth factors (PDGFs) and fibroblast growth factors (FGFs) are the most robust in their ability to induce a contractile-to-proliferative phenotype switch, and to control smooth muscle cell growth and proliferation^3^,^4^,^5^,^6^. PDGFs were one of the first growth factors identified on the basis of their ability to stimulate growth and migration of fibroblasts, SMCs, and glial cells^7^. Inhibition of SMC PDGF receptor beta (PDGFR*β*) signaling diminishes neointimal hyperplasia in balloon-injured and atherosclerotic arteries, as well as in autologous vein grafts^8^,^9^,^10^. The FGFs comprise a 22-member family of proteins that mediate their biological activity by binding to four cell surface receptor tyrosine kinases, designated FGF receptors (FGFR1-FGFR4). This results in assembly of a ternary complex that involves an FGF receptor adaptor protein fibroblast growth factor receptor substrate 2 (FRS2), Grb2 and Sos^11^ which leads to activation of the Ras/mitogen-activated protein kinase (MAPK) signaling cascade. Similar to PDGFs, FGFs promote SMCs conversion to the proliferative phenotype and stimulate their growth and migration^12^.

In contrast to FGFs and PDGFs, another growth factor, transforming growth factor beta (TGF*β*), mediates a SMC switch from the synthetic to contractile phenotype^13^,^14^. TGF*β* exerts its effects by binding and activating the TGF beta type 2 receptor (TGF*β*R2). The activated TGF*β*R2 then associates with and activates TGF beta type 1 receptor (TGF*β*R1) leading to phosphorylation of downstream targets Smad2 and Smad3, resulting, eventually, in expression of SMC contractile proteins^15^,^16^,^17^,^18^. The TGF*β* pathway is critical for vascular development and homeostasis of the adult vasculature. Selective deletions of *Tgfbr1* or *Tgfbr2* in SMCs cause embryonic lethality due to vascular deformities^19^. Adult mice lacking *Tgfbr2* in VSMCs develop vascular defects including aortic wall thickening, dilatation, and dissection^20^.

A number of studies reported the ability of FGFs to antagonize TGF*β*-mediated induction of SMC markers expression in pericytes and smooth muscle cells^21^,^22^. FGFs achieve this effect by repressing expression of TGF*β* family^23^. *In vitro*, inhibition of FGF signaling upregulates TGF*β*R1 and downstream Smad2/3 activity while *in vivo* it leads to reduced SMC proliferation^23^. These observations suggest that FGF signaling input may regulate the TGF*β*-dependent proliferative to contractile SMC phenotype switch. In this study, we set out to test this hypothesis *in vitro* and *in vivo*. We found that deletion of *Frs2α* in SMC increased SMC TGF*β* signaling. This led to a proliferative to contractile phenotype conversion even in the presence of high serum (*in vitro*) and blood vessel wall injury (*in vivo*). These results support the notion that FGF-TGF*β* signaling antagonism is the key regulator of the SMC phenotype switch; thus, manipulation of this cross-talk may be an effective strategy for treatment of SMC-proliferation related diseases.

## Results

### FGF signaling is downregulated and TGF*β* signaling activity is upregulated during smooth muscle cell differentiation

**Figure 1:**
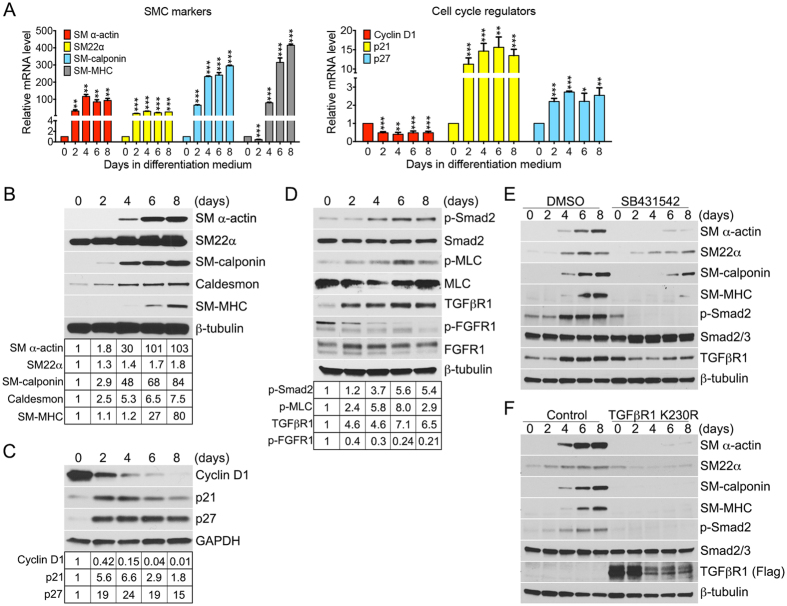
FGF activity is downregulated and TGF*β* signaling is upregulated during primary human aortic smooth muscle cell (HASMC) differentiation. HASMCs, cultured in the growth medium (M231 + SMGS), were switched to the differentiation medium (M231 + SMDS) at day 0 and cultured for 8 days (**A**) qRT-PCR analysis of SMC contractile marker expression and cell cycle regulators. *β*-actin was used for sample loading normalization. Data represent mean ± SD (*P < 0.05, **P < 0.01, ***P < 0.001 compared to control; unpaired two-tailed Student’s t test). Histogram of qRT-PCR results are representative of three independent experiments. (**B**–**D**) Upper panels: Immunoblot analysis of smooth muscle markers, cell cycle regulators (Cyclin D1, p21, p27), FGF, and TGF*β* pathways in HASMCs. Blots are representative of five independent experiments. Bottom panels: Band intensities of SM *α*-actin, SM22*α*, SM-calponin, Caldesmon, SM-MHC, Cyclin D1, p21, p27, p-Smad2, p-MLC, TGF*β*R1, and p-FGFR1 were normalized to *β*-tubulin, GAPDH, Smad2, or MLC and expressed as a fraction of a control value. (**E**–**F**) Immunoblots of SMC contractile markers, phosphorylated Smad2 (p-Smad2), and TGF*β*R1 in control, SB431542, or TGF*β*R1 K230R lentiviruse treated HASMCs. Blots are representative of three independent experiments.

Primary human aortic smooth muscle cells (HASMCs) rapidly lose expression of contractile SMC proteins when cultured in the high serum (“growth”) medium, but retain their expression in the low-serum (“differentiation”) medium^24^. As expected, switching cultured HASMC from the growth to differentiation medium resulted in a gradual increase in expression of smooth muscle contractile markers (SM *α*-actin, SM22*α*, SM-calponin, SM-MHC) and cell cycle inhibitors (p21 and p27), while the expression of the proliferative marker Cyclin D1 gradually declined (Fig. 1A). Western blot analysis demonstrated increased expression levels of SMC contractile markers (SM *α*-actin, SM22*α*, SM-calponin, caldesmon, SM-MHC) and cell cycle inhibitor proteins p21 and p27 (Fig. 1B,C), which was consistent with the qRT-PCR data.

Activation of TGF*β* signaling has been linked with the induction of SMC differentiation and cell cycle exit^13^,^25^. Therefore, we examined this signaling cascade in HASMC during their switch from growth to differentiation. There was a gradual increase in TGF*β*R1 protein expression and activation of TGF*β* signaling (increased p-Smad2 and p-MLC) (Fig. 1D), beginning on day 2 after the medium switch. There was a simultaneous decrease in FGFR1 activity, as measured by FGFR1 tyrosine kinase phosphorylation, while its expression remained unchanged (Fig. 1D). The downregulation of FGF signaling and upregulation of TGF*β* activity during smooth muscle cell differentiation process was further confirmed in mouse vascular smooth muscle cells (VSMCs) isolated from the mouse aorta (Supplementary Fig. 1). We used two different experimental approaches to test if TGF*β* activity is required for SMC differentiation. First, treatment of HASMCs with the TGF*β*R1 kinase inhibitor SB431542 during differentiation induction, significantly decreased SM *α*-actin, SM22*α*, SM-calponin, and SM-MHC expression compared to DMSO-treated controls (Fig. 1E). Second, transduction of HASMCs with a dominant negative TGF*β*R1 construct (TGF*β*R1 K230R) led to an almost a complete block of differentiation (Fig. 1F).

### FGF signaling inhibits TGF*β* activity and promotes smooth muscle cell proliferative phenotype induction

**Figure 2:**
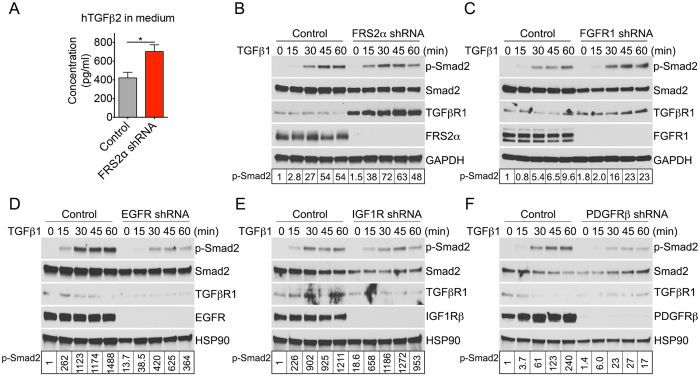
Inhibition of FGF signaling increases TGF*β* activity. HASMCs were cultured in the growth medium (M231 + SMGS). (**A**) ELISA quantification of TGF*β*2 present in the extracellular culture medium of control and FRS2*α* knockdown HASMCs. Data represent mean ± SD (*P < 0.05 compared to control; unpaired two-tailed Student’s t test. N = 3). (**B**–**F**) Upper panels: Immunoblot analysis of TGF*β* signaling in control, EGFR, FGFR1, FRS2*α*, IGF1R, and PDGFR*β* knockdown HASMCs. HASMCs were serum starved for 8 hr then stimulated with TGF*β*1 (0.5 ng/ml) for different time points. Blots are representative of three independent experiments. Bottom panels: Band intensities of p-Smad2 were normalized to Smad2 and expressed as a fraction of a control value.

Recent studies demonstrated that FGFs regulate both endothelial cell and SMC TGF*β* signaling^23^,^26^. FGF signaling input was inhibited by a short hairpin RNA (shRNA) to reduce expression of FRS2*α*, the key adaptor molecule involved in FGF receptor signal transduction^27^. Knockdown of FRS2*α*, in HASMCs cultured under growth conditions, led to increased expression of TGF*β*-related genes (Table 1) and increased expression of TGF*β*2 protein in the extracellular medium (Fig. 2A). Western blotting experiments confirmed the increased TGF*β*R1 expression and activation of TGF*β* signaling (p-Smad2) (Fig. 2B). Confocal microscopy imaging showed a strong nuclear staining for p-Smad2/3 after FRS2*α* knockdown compared to control cells following TGF*β*1 stimulation (Supplementary Fig. 2). FGFR1 is the most abundant FGF receptor in smooth muscle cells^23^. Knockdown of FGFR1 was associated with an increase in TGF*β*1-induced Smad2 phosphorylation and TGF*β*R1 expression (Fig. 2C), similar to FRS2*α* knockdown (Fig. 2B).

**Figure 3:**
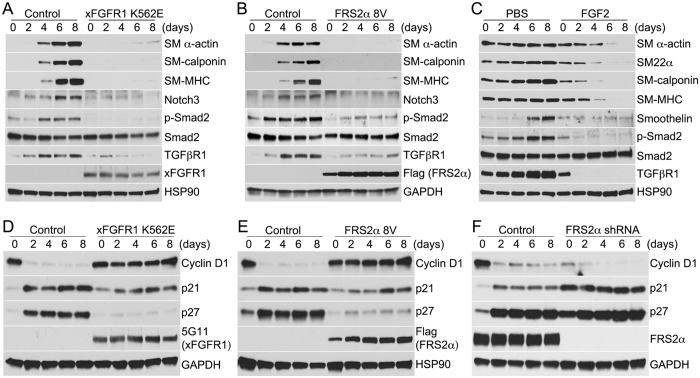
FGF signaling inhibits smooth muscle cell differentiation and induces a contractile to synthetic phenotypic switch in primary human aortic smooth muscle cells (HASMCs). (**A**,**B**) Control, constitutively active FGFR1 (xFGFR1 K562E), or constitutively active FRS2*α* (FRS2*α* 8V) lentivirus transduced HASMCs were cultured in the growth medium (M231 + SMGS) and then switched (day 0) to the differentiation medium (M231 + SMDS) for 8 days. Immunoblots of SMC contractile markers, phosphorylated Smad2 (p-Smad2), and TGF*β*R1 in control, xFGFR1 K562E, or FRS2*α* 8V overexpressed HASMCs. Blots are representative of three independent experiments. (**C**) HASMCs were cultured in the differentiation medium (M231 + SMDS) for 8 days and were then treated with PBS or FGF2 (50 ng/ml) for additional 8 days in the same medium. Immunoblots of SMC contractile markers, phosphorylated Smad2 (p-Smad2), and TGF*β*R1 in PBS and FGF2 treated HASMCs. Blots are representative of three independent experiments. (**D–F**) HASMCs were cultured in the growth medium (M231 + SMGS) at day 0 then were switched from growth medium to differentiation medium (M231 + SMDS) for 8 days. Immunoblots of Cyclin D1, p21, and p27 expression in control, xFGFR1 K562E, FRS2*α* 8V, and FRS2*α* shRNA lentivirus treated HASMCs. Blots are representative of three independent experiments.

Importantly, the effect of FGF signaling inhibition on TGF*β* activity was specific. Knockdowns of epidermal growth factor receptor (EGFR), insulin-like growth factor-1 receptor (IGF1R), and platelet-derived growth factor receptor beta (PDGFR*β*) had little effect on TGF*β*R1 protein expression or Smad2 phosphorylation in response to TGF*β*1 treatment (Fig. 2D–F). Since TGF*β* has been reported to directly regulate expression of vascular SMC differentiation markers^28^,^29^,^30^,^31^, we next examined if FGF/TGF*β* cross-talk affects this process. To this end, we used xFGFR1 K562E and FRS2*α* 8V mutant constructs which have been shown to constitutively activate FGF signaling in the absence of ligand stimulation^5^,^32^. Overexpression of either xFGFR1 K562E or FRS2*α* 8V completely suppressed growth arrest-induced SMC differentiation. This resulted in a decrease in TGF*β*R1 levels, p-Smad2 activity, and smooth muscle marker gene expression (SM *α*-actin, SM-calponin, SM-MHC, Notch3) (Fig. 3A,B). Addition of FGF2 to HASMC, cultured under growth arrest conditions, also blocked their differentiation (Fig. 3C). To further assess the role of FGF signaling in the cell cycle regulation, HASMCs were transduced with control, xFGFR1 K562E, or FRS2*α* 8V lentiviruses under growth conditions and then switching to the differentiation medium for 1 to 8 days. Increased FGF signaling activity upregulated Cyclin D1 while downregulating p21 and p27 expression (Fig. 3D,E). Conversely, inhibition of FGF signaling by a knockdown of the adaptor protein FRS2*α*, further increased p21 and p27 protein expression compared to controls (Fig. 3F). Thus, inhibition of FGF signaling overrides the proliferative phenotype-inducing effect of the growth medium while FGF stimulation overrides the contractile phenotype-inducing effects of the differentiation medium.

### Blockade of SMC FGF signaling in mice decreases neointima formation in the carotid artery ligation model

**Figure 4:**
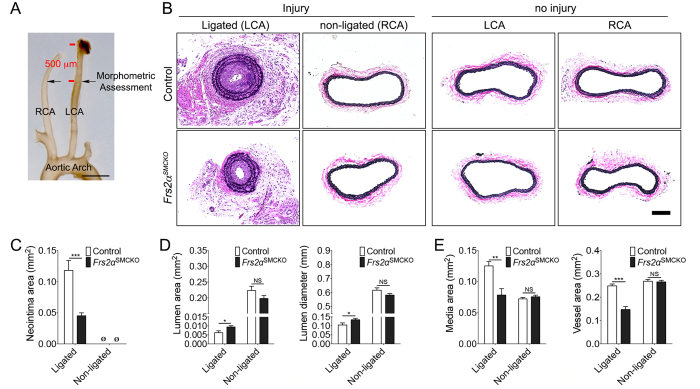
Smooth muscle cell *Frs2*α knockout inhibits neointima formation following carotid artery ligation. (**A**) Schematic of carotid anatomy and the ligation model. The left carotid artery was ligated and morphometric assessment of the left and right carotid arteries was performed after 4 weeks. RCA: right common carotid artery; LCA: left common carotid artery. (**B**) Representative images of elastic-Van Gieson (EVG) staining in ligated, non-ligated control, and no injured carotid arteries. 10 mice per group. Scale bar: 200 *μ*m. (**C**–**E**) Quantification of the neointima, lumen, media, vessel areas, and lumen diameter from the ligated and non-ligated arteries. (∅: not detected, NS: not significant, *P < 0.05, **P < 0.01, ***P < 0.001 compared to control, unpaired two-tailed Student’s t test).

To investigate whether FGF signaling affects SMC differentiation and phenotype switch *in vivo*, we used a recently reported mouse line with the SMC deletion of *Frs2α*^23^. The carotid ligation model was used to assess the effect of this deletion in the *Frs2α*^SMCKO^ and control mice on smooth muscle proliferation and vascular remodeling. In this blood-flow cessation model, after an early phase of inflammatory cell recruitment, medial SMCs rapidly proliferate and migrate toward the lumen. This results in extensive neointima formation four weeks later (Fig. 4A)^33^. Morphometric analysis of carotid arteries 4 weeks after the ligation demonstrated a marked decrease in the amount of neointima (62% reduction) and an increase in lumen area (51%) and lumen diameter (28%) in the left (ligated) carotid arteries of *Frs2α*^SMCKO^ compared to control mice (Fig. 4B–D). In addition, there was a significant decrease in vessel remodeling of ligated arteries in *Frs2α*^SMCKO^ mice. This resulted in smaller media and total vessel areas in the ligated arteries of *Frs2α*^SMCKO^ mice (Fig. 4E). There were no significant differences between non-ligated (right) carotids in the two groups.

**Figure 5:**
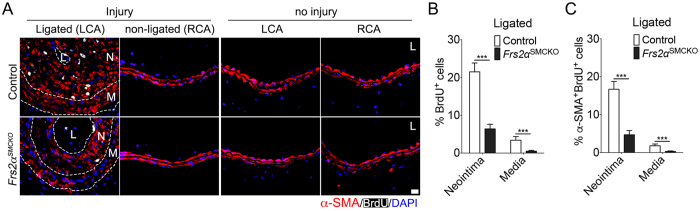
Smooth muscle cell *Frs2α* knockout inhibits cell proliferation following carotid artery ligation. (**A**) Immunofluorescence staining for *α*-SMA (red) and BrdU (white) in ligated, non-ligated control, and no injured carotid arteries. Nuclei were stained with DAPI (blue). Images are representative of 10 mice per group. L, lumen; N, neointima; M, media. Scale bar: 16 *μ*m. (**B,C**) Quantification of the number of neointima and medial smooth muscle cells expressing BrdU and *α*-SMA (***P < 0.001 compared to control, unpaired two-tailed Student’s t test).

SMC proliferation is a major contributor to neointima formation in this model^33^. We used 5-Bromo-2′-deoxyuridine (BrdU) to assess its extent over the 2 week time course. To this end, BrdU was injected subcutaneously every other day for 2 weeks starting 2 weeks after the ligation. Quantification of the BrdU^+^ signal revealed that in control mice, 21.5% of cells in the neointima and 3.4% of cells in the media of control mice stained positive for BrdU 28 days post-ligation, while 6.4% of the neointimal SMCs and 0.5% of medial SMCs were BrdU positive in *Frs2α*^SMCKO^ mice (Fig. 5A,B). Similarly, quantification of the *α*-SMA^+^BrdU^+^ signal revealed that 16.7% of cells in the neointima and 1.8% of cells in the media of control mice stained positive for *α*-SMA^+^BrdU^+^ 28 days post-ligation, while 4.7% of the neointimal SMCs and 0.3% of medial SMCs were BrdU positive in *Frs2α*^SMCKO^ mice (Fig. 5A,C). BrdU^+^ labeling was not detected in contralateral non-ligated arteries and in uninjured arteries in control and *Frs2α*^SMCKO^ mice (Fig. 5A).

## Discussion

This report provides several lines of evidence to support the hypothesis that FGF-TGF*β* signaling cross-talk is the key regulator of vascular SMC phenotype modulation. Suppression of FGF signaling in primary SMC *in vitro* activated TGF*β* signaling and induced SMC differentiation, even in the presence of a high concentration of serum. At the same time, activation of FGF signaling inhibited TGF*β* activity and induced conversion to the proliferative phenotype, even when cells were cultured under the low serum (differentiation medium) conditions. This was further verified *in vivo* where SMC deletion of *Frs2α* induced TGF*β* signaling and prevented normal contractile media SMC from acquiring a proliferative phenotype, even in the presence of severe vascular injury (carotid artery ligation). The end result was a marked reduction in medial SMC proliferation and a decrease in the neointima formation.

Interestingly, *Frs2α*^SMCKO^ mice were viable and born at the expected Mendelian frequency. This suggests that the absence of FGF signaling in smooth muscle cells can be compensated by other signaling pathways during development, maturation, and adult homeostasis^23^. Many growth factors are capable of inducing SMC contractile to synthetic phenotype switch, which stimulates SMC growth and migration. However, FGF regulation of TGF*β* signaling in this context is unique. That is, activation of TGF*β* activity, due to reduced FGF signaling input, could not be overridden by high serum concentration *in vitro* or by vascular injury *in vivo*; thus, SMC remained in the contractile state. Similarly, FGF stimulation of growth arrested SMCs induced a proliferative phenotype by suppressing TGF*β* signaling in the absence of any other growth factor.

The biological significance of this cross-talk is demonstrated by the *in vivo* results. Smooth muscle cells in the arterial media, in the absence of disease or injury, are found in the G0/G1 phase of the cell cycle. They exhibit high expression levels of contractile smooth muscle marker genes. SMC attain a synthetic state in response to vascular injury, characterized by a high rate of cell proliferation, migration, and production of extracellular components. This leads to formation of neointima, a feature of a number of vasculo-proliferative conditions ranging from atherosclerosis to restenosis^1^,^2^,^33^. This switch to the synthetic (proliferative) phenotype is thought to be triggered by changes in local environmental cues, including an increase in the local concentration of mitogens such as EGF, IGF, PDGF, and FGFs. Previous studies showed that inhibition of FGF signaling by an anti-FGF2 antibody or an FGFR tyrosine kinase inhibitor SU5402, resulted in decreased SMC proliferation and attenuation of neointimal thickening^4^,^34^,^35^. However, given the broad nature the anti-FGF2 antibody or FGFR inhibitor activity, the mechanism of these effects remained elusive. The results of our study indicate that it is the SMC FGF-TGF*β* cross-talk that is central to these effects. The nature of FGF-dependent regulation of TGF*β* signaling is related to the previously demonstrated FGF dependence of *let-7* miRNA expression^23^. The *let-7* miRNA binds to TGF*β*R1 3′UTR and inhibits TGF*β*R1 expression at mRNA levels^26^. Thus, changes in *let-7* levels have a profound effect on TGF*β*R1 expression. The decline in *let-7* levels in the absence of the FGF signaling input activates TGF*β* signaling while the opposite is true during FGF stimulation.

It is important to note that SM22*α*-Cre activity is not limited to SMC and it is also expressed in cardiomyocytes and in myeloid cells^36^,^37^. Bone marrow-derived cells contribute to neointima formation^38^,^39^,^40^. Therefore, we cannot definitively rule out the possibility that the decreased neointima formation in *Frs2α*^SMCKO^ mice was due, in part, to the effect of FGF-TGF*β* cross-talk in myeloid cells. This issue can be resolved using a more SMC specific Cre such as *Myh11*-CreER^T2^ 20,41, and will be the subject of future studies. This study also provides an interesting insight into the origin of neointimal SMCs. Several sources for these cells have been proposed, including blood and bone marrow derived precursor cells^38^,^39^,^40^, dedifferentiated medial VSMCs, resident progenitor cells, adventitial fibroblasts^42^,^43^,^44^ and the endothelium due to endothelial-to-mesenchymal transition (EndMT)^26^,^45^,^46^. Importantly, there is little quantitative information regarding contribution of each of these potential sources. In the current study we observed a dramatic reduction in medial SMC proliferation after carotid ligation in *Frs2α*^SMCKO^ mice. Indeed, there was essentially no BrdU-positive cells in the media of these animals. This translated in to a 62% reduction in the neointima size. This implies that ~40% of the neointima in this model forms in a non-SMC, non-myeloid cell-dependent manner. Endothelial-to-mesenchymal transition likely accounts for the lion share of these cells^45^,^47^. In summary, this study provides *in vitro* and *in vivo* evidence that FGF signaling is the primary controller of SMC phenotype modulation and that this control is achieved via direct regulation of TGF*β* activity.

## Methods

### Growth factors and Chemicals

Recombinant human FGF2 (R&D 233-FB-001MG/CF) and recombinant human TGF*β*1 (BioLegend 580702) were reconstituted in 0.1% BSA/PBS. The TGF*β*R1 kinase inhibitor SB431542 (Sigma S4317) was reconstituted in DMSO (Sigma D2650) and used at a final concentration of 10 *μ*M in cell culture.

### Antibodies

The following antibodies were used for immunoblotting (IB), immunofluorescence (IF), or immunohistochemistry (IHC): BrdU (abcam ab6326; IHC 1:100), Calponin (Sigma C2687; IB 1:1000 for human cells), Calponin (abcam ab46794; IB 1:1000 for mouse cells), Caldesmon (Sigma C4562; IB 1:1000), Cyclin D1 (Santa Cruz sc-20044; IB 1:1000), EGFR (Cell Signaling #4267; IB 1:1000), FGFR1 (phospho Y654) (abcam ab59194; IB: 1:1000), xFGFR1 (5G11; IB 1:50)^48^, Flag (Sigma F1804; IB 1:1000), FRS2 (Santa Cruz sc-8318; IB 1:1000), GAPDH (glyceraldehyde phosphate dehydrogenase) (Cell Signaling #2118; IB 1:1000), HSP90 (Sigma 4300541; IB 1:1000), IGF-1 Receptor (Cell Signaling #3027; IB 1:1000), myosin (smooth) (Sigma M7786; IB 1:1000 for human cells), phospho-myosine light chain 2 (Thr18/Ser19) (Cell Signaling #3674; IB 1:1000), myosine light chain 2 (Cell Signaling #3672; IB 1:1000), Notch3 (abcam ab23426; IB 1:1000), p21 (Cell Signaling #2947; IB 1:1000 for human cells), p21 (abcam ab109199; IB 1:1000 for mouse cells), p27 (Cell Signaling #3688; IB 1:1000), PDGFR*β* (Cell Signaling #3169; IB 1:1000), SM22*α* (abcam ab14106; IB 1:2000), phospho-Smad2 (Ser465/467) (Cell Signaling #3108; IB 1:1000), phospho-Smad2 (Ser465/467)/Smad3 (Ser423/425) (Cell Signaling #8828; IF 1:200), Smad2 (Cell Signaling #3122; IB 1:1000), Smad2/3 (BD 610843; IB 1:1000), smooth muscle *α*-actin (Sigma A2547; IB 1:2000, IHC 1:400), smooth muscle myosin heavy chain 11 (abcam ab53219; IB 1:1000 for mouse cells), smoothelin (Millipore MAB3242; IB 1:1000), TGF*β*R1 (Santa Cruz sc-398; IB 1:1000), and *β*-tubulin (Sigma T7816; IB 1:2000).

### Cell culture and reagents

Human 293T T17 cells (human embryonic kidney cells, ATCC CRL-11268) were maintained in Dulbecco’s modified Eagle’s medium (Gibco 11965-092) with 10% fetal bovine serum (Life Technologies 16000-044) and penicillin-streptomycin (15140-122, Gibco), and were grown at 37 °C, 5% CO2. Human aortic SMCs (#C-007-5C), media (#M231-500), and supplements (SMGS: S-007-25; SMDS: S-008-5) were purchased from Life Technologies. The cells were grown at 37 °C, 5% CO2 in Medium 231 supplemented with smooth muscle growth supplement (4.9% FBS, 2 ng/ml FGF2, 0.5 ng/ml EGF, 5 ng/ml heparin, 2 *μ*g/ml IGF-1, and 0.2 *μ*g/ml BSA). For SMC differentiation, HASMC were incubated with Medium 231 containing smooth muscle differentiation supplement (1% FBS and 30 *μ*g/ml heparin) for different lengths of time. Primary human aortic SMCs between passages 6 and 10 were used in all experiments. Primary mouse SMCs were isolated from mouse aorta as previously described^20^. In brief, aortas were predigested for 5 minutes at 37 °C in HBSS (Gibco 14170-112) solution containing 1 mg/ml collagenase type A (Sigma 10103578001) to facilitate removal of the adventitia under a dissecting microscope. The denuded vessels were transferred into 3 ml HBSS solution containing 2 mg/ml collagenase type A and 0.5 mg/ml elastase (Worthington LS006365) and incubated at 37 °C for 30 minutes. Digestion was stopped with growth medium, the mixture was centrifuged, and the cells were resuspended in Claycomb medium (Sigma 51800C) supplemented with 10% fetal bovine serum (Life Technologies 16000-044), 10 units/ml Penicillin/10 *μ*g/ml Strep (Gibco 15140-122), 2 mM L-glutamine (Gibco 25030-081), and cultured in 35 mm dishes. For mouse SMC differentiation, the cells were maintained in Dulbecco’s modified Eagle’s medium (Gibco 11965-092) with 0.5% fetal bovine serum (Life Technologies 16000-044). Primary mouse SMCs between passages 2 and 3 were used in all experiments.

### Generation of lentiviruses

Human EGFR, FGFR1, IGF1R, and PDGFR*β* shRNA lentiviral constructs were purchased from Sigma while human FRS2*α* shRNA lentiviral construct was purchased from Open Biosystems. For the production of shRNA lentivirus, 3.7 *μ*g of Δ8.2, 0.2 *μ*g of VSVG, and 2.1 *μ*g of pLKO.1 carrying the control, EGFR, FGFR1, FRS2*α*, IGF1R, or PDGFR*β* shRNAs were co-transfected into 293T cells using X-tremeGENE 9 DNA Transfection Reagent (Sigma 6365787001). Forty-eight hr later the medium was harvested, cleared by 0.45 *μ*m filter (PALL Life Sciences 4184), mixed with polybrene (5 *μ*g/ml) (Sigma H9268), and applied to cells. After 6 hr of incubation, the virus-containing medium was replaced by the fresh medium. For the production of xFGFR1 K562E^5^, FRS2*α* 8V^32^, or TGF*β*R1 K230R^26^, 10 *μ*g of pLVX-IRES carrying the xFGFR1 K562E, FRS2*α* 8V, or TGF*β*R1 K230R, 5 *μ*g of pMDLg/PRRE, 2.5 *μ*g of RSV-REV, and 3 *μ*g of pMD.2G were co-transfected into 293T cells using X-tremeGENE 9 DNA Transfection Reagent (Sigma 6365787001). Forty-eight hr later, the medium was harvested, cleared by 0.45 *μ*m filter (PALL Life Sciences PN4184), mixed with 5 *μ*g/ml polybrene (Sigma H9268), and applied to the cells. After 6 hr incubation, the virus-containing medium was replaced by fresh medium.

### RNA isolation, qRT-PCR, and gene expression profiling

Cells were suspended in TRIzol Reagent (Invitrogen #15596018), and total RNA (QIAGEN #74134) was isolated according to the manufacturer’s instructions. Reverse transcriptions were performed by using iScript cDNA synthesis kit (Bio-Rad 170-8891). qRT-PCR was performed using Bio-Rad CFX94 (Bio-Rad) by mixing equal amount of cDNAs, iQ SYBR Green Supermix (Bio-Rad 170-8882) and gene specific primers SABiosciences (a QIAGEN company) (ACTB [PPH00073G], CCND1 [PPH00128F], MYH11 [PPH02469A], CDKN1A [PPH00211E], CDKN1B [PPH00212C], ACTA2 [PPH01300B], TAGLN [PPH19531F], and CNN1 [PPH02065A]. All reactions were done in a 20 *μ*l reaction volume in duplicate. Individual mRNA expression was normalized in relation to expression of endogenous *β*-actin. PCR amplification consisted of 5 min of an initial denaturation step at 95 °C, followed by 46 cycles of PCR at 95 °C for 15 s, 60 °C for 30 s. For RT^2^ Profiler PCR Array, Human TGF*β*/BMP Signaling Pathway (QIAGEN PAHS-035ZA) RT^2^ Profiler PCR Arrays were used following the manufacturer’s protocol. Each array plate contains five commonly used human housekeeping genes (ACTB, B2M, GAPDH, HPRT1, and RPL13A), and a panel of proprietary control genes to monitor genomic DNA contamination (HGDC) as well as the first strand synthesis (RTC) and real-time PCR efficiency (PPC). The plate was subjected to real-time PCR with a two-step cycling program in a Bio-Rad CFX96 System: 95 °C for 10 min, followed by 40 cycles of 95 °C for 15 sec and 60 °C for 1 min. The resulting threshold cycle values for all wells were exported to a blank Excel spreadsheet, and then uploaded to the SABiosciences Web for data analysis using the SABiosciences Web-based PCR Array Data Analysis Software version 3.5 (http://www.sabiosciences.com/pcr/arrayanalysis.php).

### TGF*β*2 content

Cultured HASMCs from control and FRS2*α* knockdown were seeded at a confluent cell density (150000 cells in 6 cm culture plate) and then maintained in culture for three days without changes of the culture medium. Thereafter, the respective extracellular medium was collected and a commercially available sandwich ELISA kit (R&D Systems #DB250) was used to evaluate the secretion of TGF*β*2 in the extracellular medium. The absorbance (450 nm) for each sample was analyzed by a microplate reader (BioTek Synergy2 multi-Mode) and was interpolated with a standard curve.

### Western Blot Analysis

Cells were lysed with HNTG lysis buffer (20 mM HEPES, pH 7.4/150 mM NaCl/10% glycerol/1% Triton-X 100/1.5 mM MgCl2/1.0 mM EGTA) containing complete EDTA-free protease inhibitors (Sigma #11836170001) and phosphatase inhibitors (Sigma #04906837001). 20 *μ*g of total protein from each sample were resolved on Criterion TGX Precast Gels (Bio-Rad #567-1084) with Tris/Glycin/SDS Running Buffer (Bio-Rad #161-0772), transferred to nitrocellulose membranes (Bio-Rad #162-0094) and then probed with various antibodies. Chemiluminescence measurements were performed using SuperSignal West Pico Chemiluminescent Substrate (Thermo Fisher Scientific Prod #34080).

### Quantification of Western blots

Images of blot signals on HyBlot ES^®^ Autoradiography Film (DENVILLE E3218) were scanned on a CanoScan LiDE 200 scanner. Images were then viewed in ImageJ software for data analysis. Signal intensities of individual bands were determined using Gel Analysis followed the ImageJ user’s guide. Data were exported to Prism 7 to generate the plot. Data are presented as fold change in protein expression for the experimental groups compared to the control group after normalized to loading controls (GAPDH, HSP90, *β*-tubulin or total phospho-protein).

### Immunofluorescence Staining

Cultured primary HASMCs were grown on 10 *μ*g/ml fibronectin (Sigma F2006) coated glass-bottomed dishes (MatTek CORPORATION P35G-1.5-20-C). Cells were first fixed with 2% paraformaldehyde (Polysciences, Inc, 18814) in PBS for 20 minutes at 37 °C then permeabilized with ice-cold 100% methanol for 10 minutes at −20 °C, and blocked with 5% normal serum/0.3% Triton X-100 at room temperature for 60 minutes. Cells were then washed with PBS and incubated with p-Smad2/3 (1:200 in 1% BSA/0.3% Triton X-100) at 4 °C overnight, washed three times with PBS and incubated with diluted Alexa Fluor-conjugated secondary antibody (1:500) (Invitrogen) for 1 hour at room temperature. The dishes were then washed three times with PBS and mounted using Prolong Gold antifade reagent with DAPI (Life technologies P36931).

### Generation of mice

All mice were maintained on a C57BL/6 background. *Frs2α*^flox/flox^ mice were previously described^49^. *Frs2α*^flox/flox^ mice were crossed with mice expressing Cre recombinase under the control of SM22*α* promoter^50^. PCR genotyping analysis was done using the following primers: *Frs2α*^flox/flox^ (5′-GAGTGTGCTGTGATTGGAAGGCAG-3′ and 5′-GGCACGAGTGTCTGCAGACACATG-3′), SM22*α*-Cre (5′-GCGGTCTGGCAGTAAAAACTATC-3′, 5′-GTGAAACAGCATTGCTGTCAC TT-3′, 5′-CTAGGCCACAGAATTGAAAGATCT-3′, and 5′-GTAGGTGGAAATTCTAGCATCATCC-3′). All animal procedures were performed under protocols approved by Yale University Institutional Animal Care and Use Committee and carried out in accordance with the approved protocol and the relevant University guidelines regarding the use of anesthesia, post-operative monitoring and performance of surgical procedures.

### Mouse carotid ligation model

To achieve carotid artery ligation, the left carotid artery of an anesthetized 8–10 week-old mouse was isolated and ligated with a 6–0 silk suture just proximal to the carotid bifurcation through a midline cervical incision (Fig. 4A)^51^. The wound was close with a 5–0 prolene suture. Four weeks after injury, all animals were anesthetized and perfused with PBS, followed by 4% paraformaldehyde for 5 min. Both left and right carotid arteries were excised and embedded in paraffin. Lumen diameter, lumen area, neointima area, media area, and total vessel area were measured using NIH Image J. Animals also received 8 subcutaneous injections of the thymidine analog 5-Bromo-2′-deoxyuridine (BrdU) (Sigma B9285; 1 mg per injection).

### Histology and Immunohistochemical staining

In ligated left common carotid arteries, cross sections from the predefined proximal distances from the ligation site (500 *μ*m) were analyzed. In non-ligated right common carotid arteries or in no injured left and right common carotid arteries, sections from 500 *μ*m distance to the bifurcation of internal and external carotid artery were analyzed. Blocks were sectioned at 5 *μ*m intervals using a Microtome. Slides were dewaxed in xylene, boiled for 20 min in citrate buffer (10 mM, pH 6.0) for antigen retrieval, and rehydrated. After washing three times with phosphate-buffered saline, tissue sections were incubated with primary antibodies diluted in blocking solution (10% BSA and horse serum in PBS) overnight at 4 °C in a humidified chamber. For BrdU staining, slides were denatured with 1.5 M HCl for 20 min prior to antibody labeling. Sections were washed three times with tris-buffered saline, incubated with appropriate Alexa Fluor-conjugated secondary antibody (1:500) (Invitrogen) for 1 hr at room temperature, washed again three times, and mounted on slides with ProLong Gold mounting reagent with DAPI (Life Technologies P36931). All immunofluorescence micrographs were acquired using a Zeiss microscope. Images were captured using Volocity software and quantifications performed using ImageJ software (NIH).

### Statistical analysis

Graphs and statistical analysis were prepared using GraphPad Prism. Data are expressed as mean ± SD. The level of statistical significance was determined by 2-tailed Student’s t test as appropriate using the GraphPad Prism software. A P value less than 0.05 was considered significant (*P < 0.05, **P < 0.01, ***P < 0.001). All results were confirmed by at least 3 independent experiments. Error bars represent mean ± SD.

## Additional Information

**How to cite this article**: Chen, P.-Y. *et al*. Fibroblast growth factor (FGF) signaling regulates transforming growth factor beta (TGF*β*)-dependent smooth muscle cell phenotype modulation. *Sci. Rep.* **6**, 33407; doi: 10.1038/srep33407 (2016).

## Supplementary Material

Supplementary Information

## Figures and Tables

**Table 1:** qRT-PCR array (ratio) of Human TGF*β*/BMP Signaling Pathway.

Gene symbol	Fold change (FRS2α shRNA/Control)	Gene symbol	Fold change (FRS2α shRNA/Control)
ACVR1	4.26	MECOM	5.62
BACVR2A	18	MYC	20.82
AMH	8	NOG	8
AMHR2	8	PDGFB	8
ATF4	5.03	RUNX1	4.53
BAMBI	36	SMAD1	9.92
BGLAP	9.32	SMAD2	6.19
BMP1	4.56	SMAD4	6.36
BMP3	8	SMAD5	13.09
BMP4	8	SMURF1	4.03
BMP5	8	STAT1	10.63
BMP6	4.17	TGF*β*2	35.75
BMP7	8	TGF*β*3	3.53
BMPER	4.92	TGF*β*I	12.91
BMPR1A	11.63	TGF*β*R1	5.9
BMPR1B	3.61	TGF*β*R2	4.17
BMPR2	21.26	TGF*β*R3	34.30
CDKN1A	83.87	TGF*β*RAP1	4.69
CDKN1B	3.18	TGIF1	11.96
CDKN2B	24.08	TNFSF10	51.63
CHRD	4.06		
COL1A1	5.66		
COL1A2	19.03		
DCN	39.4		
DLX2	39.67		
EMP1	44.63		
FOS	3.2		
FST	75.06		
GADD45B	26.35		
GDF2	8		
GDF3	8		
GDF5	4.89		
GDF6	8		
GDF7	8		
GSC	8		
HERPUD1	19.03		
GDF6HIPK2	22.94		
ID1	18.38		
ID2	15.67		
IFRD1	24.59		
IGF1	23.43		
IGFBP3	8.57		
IL6	64		
INHA	2.93		
INHBA	74.54		
INHBB	8		
LEFTY1	11.88		
LTBP1	10.78		
LTBP2	3.71		
