# Supplementary material for: Fibroblast growth factor (FGF) signaling regulates transforming growth factor beta (TGFβ)-dependent smooth muscle cell phenotype modulation
Source: Sci Rep. 2016 Sep 16;6:33407. doi: 10.1038/srep33407 (PMC5025753; doi:10.1038/srep33407)
Supplement: Supplementary Information [file srep33407-s1.pdf]

Fibroblast growth factor (FGF) signaling regulates transforming growth factor beta (TGFβ)-dependent smooth muscle cell phenotype modulation

Pei-Yu Chen, Lingfeng Qin, Guangxin Li, George Tellides, and Michael Simons

Supplementary Figures:

Supplementary Figure S1

Supplementary Figure S2

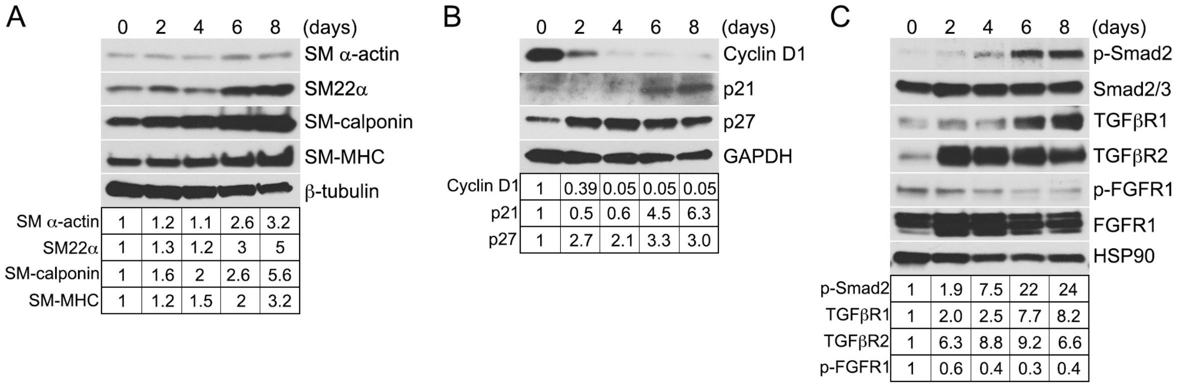

Supplementary Figure 1: FGF activity is downregulated and TGFβ signaling is upregulated during primary mouse vascular smooth muscle cell (VSMC) differentiation.

Mouse VSMCs were cultured in the growth medium (Claycomb + 10% FBS) at day 0 then were switched from growth medium to differentiation medium (DMEM + 0.5% FBS) for 8 days. (A-C) Upper panels: Immunoblot analysis of smooth muscle markers, cell cycle regulators (Cyclin D1, p21, p27), FGF, and TGFβ pathways in mouse VSMCs. Blots are representative of three independent experiments. Bottom panels: Band intensities of SM α-actin, SM22α, SM-calponin, SM-MHC, Cyclin D1, p21, p27, p-Smad2, TGFβR1, TGFβR2, and p-FGFR1 were normalized to β-tubulin, GAPDH, HSP90, Smad2, or FGFR1 and expressed as a fraction of a control value.

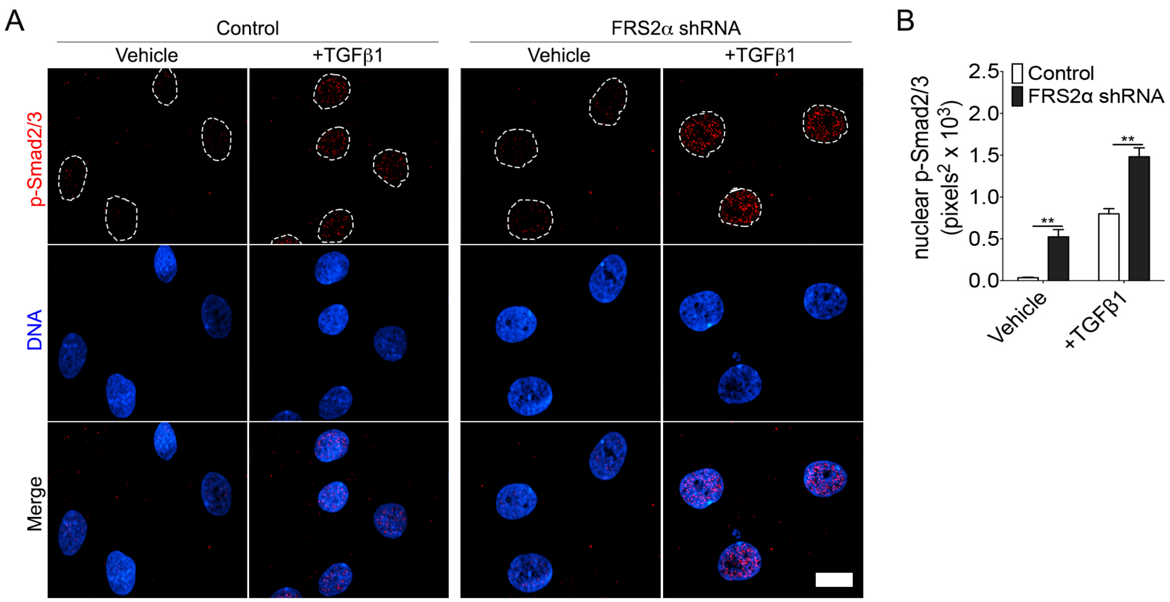

Supplementary Figure 2: FRS2α knockdown increases TGFβ signaling in primary human aortic smooth muscle cells (HASMCs).

Immunofluorescence staining of phosphorylated Smad2/3 (red) in control and FRS2α knockdown HASMCs. Nuclei were counterstained with DAPI (blue). Scale bar: 12 μm. Control and FRS2α knockdown HASMCs were serum starved for 8 hr then stimulated with TGFβ1 (0.5 ng/ml) for 15 min. Images are representative of three independent experiments.
